# Septins: New Microtubule Interacting Partners

**DOI:** 10.1100/tsw.2008.87

**Published:** 2008-06-13

**Authors:** Rosalind Silverman-Gavrila, Lorelei Silverman-Gavrila

**Affiliations:** ^1^ University of Toronto, Faculty of Medicine andToronto General Hospital, Division of Cellular and Molecular Biology, Max Bell Research Centre, Toronto, Ontario, Canada; ^2^ University of Toronto, Faculty of Medicine, Department of Physiology, Toronto, Ontario, Canada

**Keywords:** septins, microtubules, chromosome division, MAPs, exocytosis, vesicle transport, cytokinesis

## Abstract

Originally characterized as regulators of cytokinesis, septins were later implicated in other cellular processes. Recent studies show that septins have a broader role in microtubule-dependent processes, such as karyokinesis, exocytosis, and maintenance of cell shape. Many members of the septin family have been shown to colocalize or interact with the microtubule cytoskeleton, suggesting that these might be general properties of septins. Septins could play an important role in regulating microtubule dynamics by interacting with microtubule-associated proteins (MAPs) that modulate microtubule stability. Being able to associate with both microtubules and actin, septins can play an important role as adaptors between the two cytoskeletons and as regulators of processes in which both actin and microtubules are involved. As septins are associated with various neurodegenerative diseases and cancer, a better understanding of the biology of septins and their interactions with microtubules is important in order to develop possible therapeutic strategies for these diseases.

### INTRODUCTION

First identified in screens for temperature-sensitive mutations that control the budding yeast cell cycle[1], septins were later named in John Pringle's laboratory to indicate their role in septation in budding yeast. Broadly conserved, they were then found in all fungal and animal cells, but not in plants and slime *Dictyostelium*[2,3].

Originally characterized as regulators of cytokinesis in budding yeast *Saccharomyces cerevisiae*, members of the septin family have been subsequently implicated in a variety of processes in fungi and animal cells: from the selection of the budding site to the control of cell cycle checkpoints, from the establishment of a diffusion barrier for proteins to the regulation of morphogenesis, from localizing chitin deposition to the regulation of kinases, from sporulation to exocytosis, and from maintenance of cell polarity to vesicle trafficking[2,3,4].

A unifying view on how septins are involved in such diverse processes is that they represent novel cytoskeletal scaffold elements that recruit and activate various components of cell signaling assembly. While the interaction of septins with actin-based cytoskeleton and membrane-related processes is better understood, less is known about the interaction of septins with microtubules (MTs). Our review addresses recent findings that emerged from studies on the interaction between septins and MTs, possible mechanisms of interaction that these studies suggest, as well as the relevance of this interaction for the role of septin in MT-based processes.

### SEPTINS AS CYTOSKELETAL ELEMENTS

Septins purify generally as hetero-oligomeric complexes composed of different subunits that may rearrange to regulate diverse functions. Purified complexes from *S. cerevisiae*[5] and *Drosophila*[6] are able to form filaments *in vitro*, and some studies suggest that these filaments are nonpolar[7]. Septin filaments can self-assemble into higher-order structures by lateral stacking and tandem annealing, by forming curved bundles, rings, and coils[8]. In negative-staining EM, septins appear as filaments 7–9 nm in width and of variable length[5]. Their periodicity varies from 26 nm in *Drosophila*[6] to 32 nm in yeast and 25 nm in rat[9].

Septins represent a conserved family of GTP binding proteins, and can associate with membranes and colocalize with actin and MT cytoskeletons. The regulation of septin filament dynamics is very complex and the exact role of GTP binding and turnover is not fully understood with respect to the assembly to filamentous septins. There are studies that show that septin polymerizes *in vitro* in a GTP-dependent manner[10] and that GTP binding is necessary for septin filament organization[11], while others show that assembly of filaments from multiseptin complexes does not depend on exogenously added guanine nucleotide[12,13].

The dependence of septin function on polymerization is also poorly understood: some groups showed that septin function depends on septin polymerization[11], while others showed that organized septin filaments are not required for septin function[5].

### SEPTINS COLOCALIZE WITH MICROTUBULES

Septins are usually associated with sites of cell division or bud formation, but they are also found at places where the cytoskeleton is dynamically rearranged, such as the sites of neuronal growth cone, the leading edge of epithelial cells, the actin-rich regions of fibroblasts cortex, or the cortex in early *Drosophila* embryos[14]. Septin interaction with actin, membrane proteins, and components of the cytokinetic apparatus are well documented, however, only recent studies showed that septins, including many of the 14 septin isoforms from mammalian cells[15], also interact with the MT cytoskeleton (Table 1).

A first indication that septins might interact with MTs was the observation that *Drosophila* septins Pnut, Sep1, and Sep2 can bind MTs *in vitro*[16]. Later on, more studies showed that other members of the septin family colocalize with MTs. SEPT1 localizes to spindle poles (at the centrosome and nearby MT) throughout mitosis, to the midbody in telophase, and the contractile ring in cytokinesis in HeLa cells[17]. SEPT1 also partially colocalizes at midbody with Aurora B kinase, a chromosomal passenger protein essential for chromosome segregation and cytokinesis, and can be phosphorylated by Aurora B kinase *in vitro*[17]. SEPT1 localization at spindle poles and its possible regulation *in vivo* by Aurora B kinase suggests that SEPT1 may function prior to cytokinesis in chromosome segregation and during cytokinesis. A similar cell cycle–dependent distribution was noted for SEPT9_v1/MSF-A (mammalian septin mixed lineage leukemia septin-like fusion A) expressed predominantly in mammary human epithelial cells[18].

**TABLE 1**
**Interaction of Mammalian Septins with the MT Cytoskeleton**

| Septin Isoforms | Interaction |
|---|---|
| SEPT1 | SEPT1 localizes in HeLa cells to spindle poles throughout mitosis and to the midbody in telophase[17]. |
| SEPT2 | SEPT2 coimmunoprecipitates with the exocyst complex and tubulin, and aligns along interphase MTs in PC12 cells[20]. |
| | SEPT2 partially colocalizes with MT network[21,22]. |
| | SEPT2 localizes to spindles of MDCK and HeLa cells[23]. |
| | SEPT2 colocalizes with polyglutamylated MT in MDCK cells[24]. |
| SEPT5 | SEPT5 as part of the human platelet circumferential band septin complexes copurifies with the platelet MT coil and tubulin[26]. |
| SEPT6 | SEPT6 localizes to spindles of MDCK and HeLa cells[23]. |
| | SEPT6 binds MAP4 and prevents its binding to MTs, reducing MT stability[27]. |
| SEPT7 | SEPT7 binds MAP4, and prevents its binding to MTs, reducing microtubule stability[27]. |
| SEPT9 | SEPT9 is associated with MTs in interphase. Its localization to the mitotic spindle during mitosis and the interzone MTs in mammary human epithelial cells is MT dependent[18]. |
| | SEPT9 colocalization with MTs during interphase and with spindles during mitosis in HeLa cells is MT dependent[19]. |
| SEPT11 | SEPT11 colocalizes with MTs in HMEC and HeLa cells, and this localization is MT dependent[25]. |

SEPT9 is associated with MTs in interphase and localizes to the mitotic spindles, but not the spindle poles during mitosis, and to bundles of MTs at the interzone in between the separated chromosomes[18]. A similar distribution was also reported for SEPT9 in HeLa cells[19]. *In vitro* SEPT9 associates directly with MTs and this binding is saturable with a $K_d$ of 0.1 $\mu M$[18]. SEPT9 binds directly to polymerized tubulin through a central region that contains guanine nucleotide–interactive motifs and this association is independent of the GTPase activity of SEPT9[18]. Interaction with MTs is important for formation of septin filament structures containing SEPT9, as MT disruption affects septin organization[18]. SEPT9 colocalizes heterogeneously with MTs during interphase in HeLa cells, suggesting that the interaction between the two is highly dynamic[19]. Transfections of various SEPT9 isoforms demonstrated that SEPT9_v1/MSF-A specifically localizes with MTs[18,19]. This localization is MT dependent, as disruption of MTs with nocodazole causes the disappearance of septin curvilinear structures, suggesting an interaction between septins and MTs[18,19].

SEPT2(Nedd5) coimmunoprecipitates with the exocyst complex and tubulin from rat brain lysate, and aligns along interphase MTs in PC12 cells[20], suggesting that this septin also might interact with MTs. In other studies, SEPT2 was shown to colocalize partially with MTs[21,22]. MT integrity is not dependent on the interaction with this septin as small interfering RNA (siRNA) for SEPT2 or overexpression of truncated fragments of SEPT2 do not affect MT organization, while septin distribution is MT dependent as it is perturbed on MT disruption with nocodazole treatment[22]. A network of short SEPT2 filaments was visualized near the kinetochores of congressing chromosomes, as well as kinetochore MT in both fixed and living samples of MDCK (Madin darby canine kidney) and HeLa cells[23]. SEPT6 also localizes to spindles in these cells[23]. Recently, Spiliotis et al. showed that SEPT2 fibers coalign with MTs and associate specifically with the polyglutamylated subset of stable MTs in MDCK, and this distribution was dependent on MT structural integrity[24].

SEPT11 colocalizes with MTs in HMEC and HeLa cells, and this localization is MT dependent as MT disruption with nocodazole affects its distribution[25]. Interestingly, there is a difference in the localization of SEPT11 in the two cell types: SEPT11 partially colocalizes with stress fibers only in HeLa cells[25], also suggesting a role for SEPT11 in these cells in both actin- and tubulin-dependent cellular events. Colocalization of other septins, such as SEPT2[18,19], with actin suggests a possible role for some septins in the cross-talk between actin and the MT cytoskeleton.

Indirect evidence suggests a close relationship between other septins and MTs. Human platelet septin SEPT5 is part of a complex with multiple septins found mainly at the platelet periphery and the platelet circumferential band that copurifies with the platelet MT coil and tubulin[26].

The above recent data show that many mammalian septins colocalize or associate with MTs (Table 1) and that, in many cases, their localization is MT dependent. In addition, some septins colocalize with both actin and MTs, suggesting a possible role in the cross-talk between actin and the MT cytoskeleton.

### REGULATION OF MICROTUBULE ORGANIZATION AND DYNAMICS BY SEPTINS

The Macara group takes a step further in investigating the interaction between septin and MTs by demonstrating that septins not only colocalize with MTs, but cytoplasmic septins also regulate MT dynamics through interaction with MAP4. They showed that recombinant SEPT2, SEPT6, and SEPT7 bind directly to MAP4 and inhibit its binding to MTs as well as the bundling of MTs[27]. Depletion of endogenous septins by siRNA causes an increase in perinuclear MTs due to increased MT stability rather than MT nucleation[27]. In addition, septin depletion increases MT resistance to nocodazole-induced depolymerization, alters MT bundling *in vitro*, and causes mitotic defects *in vivo*; while suppression of septin expression in HeLa cells increases MT stability[27]. These data suggest that by binding and sequestering MAP4, cytoplasmic septin complexes may regulate MT dynamics, which is important for the progress through cell division, a process that requires an ample remodeling of the MT cytoskeleton.

The region that blocks the ability of the MAP4 fragment to bind and bundle MTs *in vitro* is a small proline-rich region in the C-terminal half of MAP4 that also binds to the SEPT2:6:7 heterotrimer complex as well as to the SEPT2 monomers[27]. Based on these data, a possible model suggests that MAP4 binds to MTs and stabilizes them (Fig. 1a), and septin binding to cytoplasmic MAP4 prevents MAP4 association with MTs, causing their depolymerization (Fig. 1b). It is also possible that the two proteins compete for similar binding sites on MTs. siRNA for SEPT7 also reduces the expression of SEPT2 and SEPT6, while knockdown of SEPT6 reduces the expression of SEPT7, suggesting that there is a coordinated regulation of the expression level of these septins[27]. Thus, it is difficult to discriminate if only one or all septins modulate the interaction of MAP4 with MTs.

Phosphorylation by MARK/Par-1 or Cdc2 kinase inhibits the interaction of MAP4 with MTs and leads to their destabilization[28]. To date, there are no data regarding whether septin also affects the protein kinases that regulate MAP4 activity. Data from Kremer et al.[27] suggest that additional factors coordinate with septins to regulate MAP4-MT interaction, as septins interfered only with one (PRD) of the two MT binding sites of MAP4.

Recently Spiliotis et al. showed that in MDCK cells, SEPT2 depletion or overexpression of MAP4-GFP, a cytosolic binding partner of MT-free SEPT2, causes the loss of polyglutamylated MT, suggesting that MT-bound SEPT2 plays an important role in the polyglutamylation of MT[24].

Ectopically, expression of SEPT9_v1 in human mammary epithelial cells (IHMEC) caused disorganization of tubulin microfilaments, as well as a higher mitotic index and defects in cytokinesis, suggesting that this septin isoform also plays a role in regulation of MT organization and MT-dependent processes[29]. Another study also showed a correlation between SEPT9_v1 expression and cancer cell resistance to MT-disrupting drugs[30].

In budding yeasts, astral MTs interact with the septin-dependent cytoskeleton at the bud neck and this interaction is facilitated by MAP Kar9[31]. MT dynamics at the bud neck are regulated by septin-dependent kinases Hsl1 and Gin4, which are required for MT catastrophe and the creation of a pulling force on the spindle that moves the spindle toward the bud neck[31](Fig. 1d).

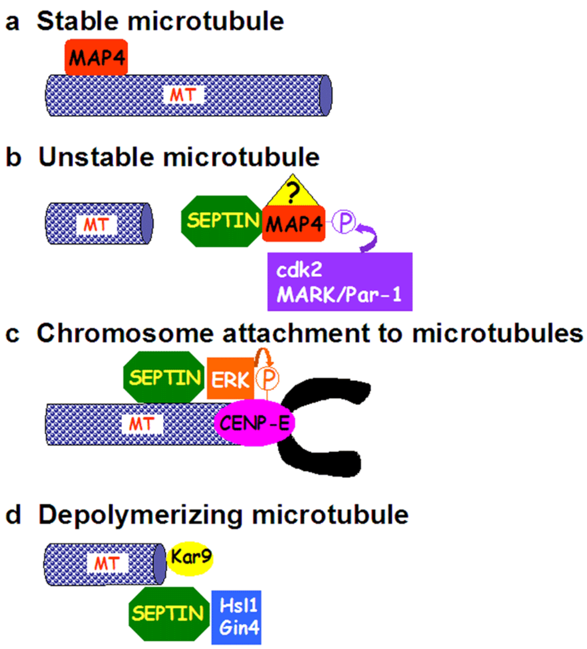

**FIGURE 1.** Possible mechanisms of regulation of the MT cytoskeleton by septins. (a,b) Septins regulate MT dynamics by regulating the activity of MAPs. (a) MT stability is enhanced when MAP4 binds to them. (b) MTs are unstable when the septin complex binds MAP4 and prevents its binding to MT. Phosphorylation of MAP4 by cdk2, MARK/Par-1, as well as possible other kinases, inhibits MAP binding to MTs. (c) Septins regulate the attachment of chromosomes to kinetochore MTs and chromosome segregation by favoring the localization of CENP-E to kinetochores and its stable attachment with the depolymerizing plus end of kinetochore MTs. Septins might also act as scaffolds for kinases that regulate CENP-E, such as Erk kinase that phosphorylates CENP-E. (d) Septins regulate the correct positioning of mitotic spindles by capturing the MT plus ends via MAP Kar9. Septin-dependent kinases Hsl1 and Gin4 are required for MT catastrophe and the creation of a pulling force on the spindle that moves the spindle toward the septin ring.

## ROLE OF SEPTINS IN MICROTUBULE-DEPENDENT PROCESSES

Colocalization of septins and MTs, and modulation of MT dynamics by septin, suggest a novel molecular function for mammalian septins. Through interaction with MT cytoskeletons, septins form a scaffold for effectors that are involved in coordinating MT-dependent cellular events, such as karyokinesis and vesicle transport along MT, and establish a connection between actin and MT cytoskeletons.

### Role of Septins in Microtubule-Based Mitotic Events

Septin association with MTs suggests a possible role in mitosis. Some septin isoforms, such as SEPT1[17], SEPT2, SEPT6[23], and SEPT9[18], localize to spindles throughout mitosis and to the midbody in telophase, and this cell cycle–dependent localization indicates that they may function in karyokinesis. Indeed, depletion of SEPT2, SEPT6, and SEPT7 increases the number of abnormal nuclei[27].

A possible role of septins in chromosome attachment to spindle MTs, in chromosome congression, and segregation is suggested by the localization of a network of short SEPT2 filaments near the kinetochores of congressing chromosomes and on kinetochore MTs[23]. Even a small decrease in the expression of SEPT2 in MDCK and HeLa cells has severe effects on cell division: it disrupts spindle MT attachment to kinetochores, causes chromosome loss from the metaphase plate, perturbs chromosome segregation and spindle elongation, and causes incomplete cytokinesis on delayed mitotic exit[23]. Reduction of SEPT2 by siRNA also affects the level of SEPT6 and SEPT7, suggesting that the observed defects might be caused by reduction of more than one septin. The defects on chromosome behavior are not secondary defects due to defects on bipolar spindle assembly, as it was shown that the tubulin level, spindle length, and the kinetochore fiber organization were not affected[23]. Interestingly, cells enter anaphase although chromosomes are misaligned at the metaphase plate, and incomplete and unstable furrows form despite abnormal chromosome segregation, suggesting that checkpoints are over-ridden on septin inactivation. Similar observations were reported after siRNA of SEPT2 that perturbs chromosome localization to the metaphase plate and cytokinesis, leading to polyploid cells[32], and after siRNA for SEPT9 that perturbs cell division and results in binucleate cells[18]. Together, these data suggest that various septin isoforms play a role in cell division in different cell types.

How septins regulate various MT-dependent events of cell division is not well known. However, recent data establish a correlation between the loss of a kinesin-like protein, the centromere-associated protein E (CENP-E), from kinetochores of mono-oriented or unattached chromosomes and septin inhibition[23]. CENP-E fails to redistribute to disassembling kinetochore spindle MTs in anaphase and to midbodies[23]. CENP-E is also required for anaphase chromosome segregation[33] and thus septins might regulate chromosome behavior from prometaphase to telophase by maintaining the proper localization and function of CENP-E at kinetochores and on spindle MTs (Fig. 1c). Septins might also act as a scaffold for kinases that regulate CENP-E, such as the MAP kinase Erk[34]. It is also possible that the checkpoint over-ride is also due to the loss of CENP-E, as CENP-E was shown to be a key factor in checkpoint signaling[35].

Additional MT-based motors or MT binding proteins might be regulated by septins in mitosis. MAP4 is one of them, as septins were shown to regulate its association with MTs[27] and thus to regulate MT dynamics, which is important for the progress through cell division. In addition, the inhibition of MT stability by septins is also important in interphase to maintain cell morphology.

The correct positioning of mitotic spindles during asymmetric cell division in budding yeast depends on the capture and stabilization of astral MTs by the septin rings that assemble at the bud neck[31]. MAP Kar9 is also involved in the capture of the MT plus end by the septin ring and MARK-related septin-dependent kinases Hsl1 and Gin4[31](Fig. 1d). Kusch et al. propose a model in which septin acts as a linker to organize and position actin- and MT-related structures relative to each other during asymmetric division in yeast[31]. However, this mechanism might be relevant to other cell systems, as septins and septin-dependent kinases are highly conserved in animal cells where MT-septin interaction could also play a role in recruitment of septins to the equator of the cell and proper positioning of the cytokinetic apparatus relative to the central spindle. Septins are also required for late stages of cytokinesis; for example, SEPT2 acts as a scaffold for myosin II and its kinases to ensure the full activation of myosin II that is necessary for the final stages of cytokinesis in mammalian cells[36]. Thus, septins have the ability to regulate both the MT and actin cytoskeleton networks during division.

In addition to cytokinesis, septins are required for normal spindle pole and centrosome organization, as inferred from the large number of multipolar spindles and extra centrosomes contained by mutants of *Drosophila* septin Pnut[37]. Localization of SEPT9 and SEPT2 to the central spindle[19] that is required for cytokinesis completion suggests that septins might also play a role in coordinating late cytokinesis events such as membrane targeting and fusion during abscission.

Colocalization of septins with spindle MTs could indicate that septins are a component of the spindle matrix whose existence was proposed decades ago and whose components play a role in spindle organization and function.

By modulating MT dynamics, by forming a regulating scaffold for mitotic proteins, and by facilitating the interaction between MT- and actin-based cytoskeletons, septins can regulate multiple steps of cell division, such as cytokinesis, spindle assembly, chromosome attachment, congression, and segregation.

#### Role of Septins in Microtubule-Based Vesicle Transport and Nervous Transmission

Another possible role of septins is in MT-dependent intracellular transport. Septins are highly concentrated in nondividing cells, such as neuronal cells, where they colocalize with MTs[37]. In neurons, the bidirectional MT-based movement of organelles and vesicles is critical for neurotransmission, synaptic plasticity, and axonal outgrowth. SEPT3 localizes predominantly to presynaptic terminals, colocalizing with synaptophysin and dynamin I[38]. SEPT3 is specifically enriched in synaptosomes and in peripheral membrane extract, and is not found in the soluble or membrane extracts[38], suggesting that SEPT3 is involved in synaptic vesicle recycling.

In neuronal cells, MTs not only play a structural role at synapses, but also serve as intracellular polar tracks for plus end–directed kinesin and minus end–directed dynein motor proteins that are necessary for the transport of mitochondria[39,40], vesicles[39], N-methyl-D-aspartate receptor (NMDAR)[41], RNA[42], etc.

Recently, Spiliotis et al. showed that septins also play a key role in the organization of MT tracks and controlling intracellular membrane transport in MDCK cells[24]. They proposed that SEPT2 binding to polyglutamylated MTs specifies a functionally distinct subset of MT tracks on which “fast track” vesicle transport occurs without the slowing down due to MAP “speed bumps”. Tubulin-associated SEPT2 facilitates vesicle transport by maintaining polyglutamylated MT tracks and impeding tubulin binding of MAP4[24], the cytoplasmic binding partner of SEPT2[27]. Vesicles containing apical or basolateral proteins exit the trans-Golgi network along SEPT2/polyGlu MT tracks, and this coupling of MT cytoskeleton to post-Golgi vesicle transport is required for the morphogenesis of polarized epithelia[24].

SEPT2 and MAP4 counteract each other in regulating vesicle transport along MTs, thus a balance between the levels of polyglutamylated MT, SEPT2, and MAP4 may control the amount and speed of vesicle transport to the plasma membrane. This regulation may be important in other cells, such as neurons, where MT-dependent vesicle transport requires polyglutamylated MT[43] and the dissociation of MAPs from MT[44].

Having the capacity to interact with both actin and MTs, as well as membranes and the exocyst complex, septins might serve as a linker of the two cytoskeletons in coordinating the distribution of organelles and vesicles to regulate neurite outgrowth, exocytosis, and synaptic transmission.

### CONCLUSIONS AND FUTURE DIRECTIONS

New insights have emerged recently on septin interaction with the MT cytoskeleton besides their more documented interaction with actin cytoskeleton and membranes. By interacting with MTs and by affecting their dynamics, their interaction with MAPs and MT-dependent motors, septins can act as modulators of multiple cellular events, such as chromosome congression, anaphase chromosome movement, chromosome attachment to spindle MT, nuclear orientation, vesicle trafficking along MTs, cell abscission, etc. Being able to interact with both actin and the MT cytoskeleton, septins can position actin- and MT-related structures relative to each other and play a pivotal role in cell division.

Septins that interact with spindle MTs have a diverse localization: closer to the plus end of MTs or near the minus end and the centrosomes, suggesting that they might regulate MT dynamics via multiple mechanisms. Thus, finding key players through which septins regulate MT dynamics and elucidating the structure, regulation, and interaction partners of septins will further our understanding of the role played by septins. Another very important field of future studies is the dissection of the role of septins in the cross-talk between actin and MT cytoskeletons, as both play an important role in many of the processes in which septins are also involved (karyokinesis, cytokinesis, cellular transport, etc.).

The study of septin interaction with the MT cytoskeleton also has a therapeutic relevance, as perturbed expression of septins that interact with MTs such as SEPT6 might contribute to resistance to chemotherapeutic drugs such as vincristine or taxol, which target MTs[45]. SEPT9_v1 expression also correlates with susceptibility of a wide range of cancer cells to 2-methoxyestradiol and paclitaxel, suggesting that SEPT9_v1 could serve as a biomarker for therapeutic resistance to MT-disrupting agents[30]. Manipulation of septins by developing inhibitory agents that specifically block them could open an avenue to therapeutics for diseases in which septins are implicated such as Alzheimer's[46], Parkinson's[47], and various types of cancer[45].

### ACKNOWLEDGMENTS

We are thankful to the Heart and Stroke Foundation and Natural Sciences and Engineering Research Council of Canada (NSERC) for postdoctoral fellowships to R. Silverman-Gavrila and L. Silverman-Gavrila, and to Dr. L. Langille, Dr. M. Charlton, and Dr. A. Wilde for their support and invaluable discussion, and to C. Falk for editing the manuscript.
